# Outer Membrane Vesicle Vaccines from Biosafe Surrogates Prevent Acute Lethal Glanders in Mice

**DOI:** 10.3390/vaccines6010005

**Published:** 2018-01-10

**Authors:** Michael H. Norris, Mohammad S. R. Khan, Sunisa Chirakul, Herbert P. Schweizer, Apichai Tuanyok

**Affiliations:** 1 Department of Infectious Diseases and Immunology, College of Veterinary Medicine; University of Florida, Gainesville, FL 32608, USA; mhnorris@ufl.edu; (M.H.N.); siddiqur.r.khan@ufl.edu (M.S.R.K.); schirakul@ufl.edu (S.C.); 2 Emerging Pathogens Institute, University of Florida, Gainesville, FL 32610, USA; hschweizer@ufl.edu; 3 Department of Molecular Genetics and Microbiology, College of Medicine, University of Florida, Gainesville, FL 32603, USA

**Keywords:** glanders, outer membrane vesicles, biosafe vaccines

## Abstract

*Burkholderia mallei* is a host-adapted Gram-negative mammalian pathogen that causes the severe disease glanders. Glanders can manifest as a rapid acute progression or a chronic debilitating syndrome primarily affecting solipeds and humans in close association with infected animals. In USA, *B. mallei* is classified as one of the most important bacterial biothreat agents. Presently, there is no licensed glanders vaccine available for humans or animals. In this work, outer membrane vesicles (OMVs) were isolated from three attenuated biosafe bacterial strains, *Burkholderia pseudomallei* Bp82, *B. thailandensis* E555, and *B. thailandensis* TxDOH and used to vaccinate mice. *B. thailandensis* OMVs induced significantly higher antibody responses that were investigated. *B. mallei* specific serum antibody responses were of higher magnitude in mice vaccinated with *B. thailandensis* OMVs compared to levels in mice vaccinated with *B. pseudomallei* OMVs. OMVs derived from biosafe strains protected mice from acute lethal glanders with vesicles from the two *B. thailandensis* strains affording significant protection (>90%) up to 35 days post-infection with some up to 60 days. Organ loads from 35-day survivors indicated bacteria colonization of the lungs, liver, and spleen while those from 60 days had high CFUs in the spleens. The highest antibody producing vaccine (*B. thailandensis* E555 OMVs) also protected C57BL/6 mice from acute inhalational glanders with evidence of full protection.

## 1. Introduction

The disease glanders is primarily a zoonotic disease of solipeds that in ancient times was recognized by both Hippocrates and Aristotle [1]. It is caused by the organism *Burkholderia mallei*, which is a host-adapted Gram-negative pathogen [2]. Historically, human cases of glanders were most often associated with individuals that were in close contact with infected horses, mules, or donkeys and was contracted through spreading of infectious secretions [3,4]. More recently, beginning in the 20th century and its two World Wars, *B. mallei* has been associated with biological warfare, intentional misuse, and laboratory acquired infections [5,6,7]. It was used as a biological weapon in World War I by Germany and the Japanese in World War II researched human infections and intentional contamination of public water sources. Following WWII, the USSR conducted field tests and anecdotal evidence suggests usage in Afghanistan. The disease has been eradicated in North America, Australia, and most of Europe through careful surveillance, culling of infected animals and importation practices [8,9]. The modern era has ushered in the global movement of livestock leading to increased cases in other parts of the world and the labeling of glanders as a re-emerging threat [10]. 

Generally, the disease itself can manifest as an acute or chronic infection. The acute disease presents as a localized cutaneous infection (also known as farcy) or pulmonary infection that can progress to either septicemia or severe pneumonic disease thereafter [4].Estimated incubation periods for acute infections may vary from 1–14 days and if left untreated can lead to death within the same period [11]. Chronic infections are indicated by chronic nasal discharge, chronically enlarged lymph nodes or lymphatic vessels, and in the case of farcy, numerous cutaneous ulcerations [2,3]. Chronic disease onset can occur up to 12 weeks after exposure, and if untreated, leads to death [4,11]. The organism is highly infectious with reports in the animal model estimating the LD_50_ at between 2 and 820 CFU depending on the strain of *B. mallei*, route of infection and rodent model utilized [9,12,13,14]. The United States Centers for Disease Control and Prevention (CDC) considers the organism as a potential threat to human health while the United States Department of Agriculture (USDA) considers it a threat to animal health and so *B. mallei* is listed as a Tier 1 overlap select agent. A vaccine against glanders is currently unavailable and treatment remains difficult. *B. mallei* shares close genetic homology to the fellow select agent *B. pseudomallei*, the etiological agent of melioidosis, and has been determined to be a species borne of *B. pseudomallei* by host-adaptation and gene loss [15,16]. *Burkholderia thailandensis* is similar to *B. pseudomallei* and *B. mallei* but is relatively avirulent with LD_50_ levels of ~10^6^ CFU in the susceptible Syrian hamster model [17,18]. High genetic homology and similar mechanisms of molecular pathogenesis within the three species mean they share numerous antigenic determinants. Important virulence factors such as the major type 3 secretion system (T3SS) *Burkholderia* secretion apparatus (Bsa) [19], type 6 secretion system 1 (T6SS-1) [20,21,22], *Burkholderia* intracellular motility protein A (BimA) [23] and lipopolysaccharide (LPS) are conserved [24,25]. *B. thailandensis* lacks the T3SS-3 that is present in *B. mallei* and *B. pseudomallei.* There are differences in O-antigen modifications of *B. mallei* LPS [26,27] and most *B. thailandensis* strains lack the major capsular polysaccharide possessed by *B. mallei* and *B. pseudomallei* [28]. Exceptions to the rule have been discovered, where environmental isolates of *B. thailandensis* strains with a capsule have been identified but remain highly attenuated [29]. A recent paper has demonstrated that CPS isolated from one such strain, (*B. thailandensis* strain E555) can afford significant protection to mice challenged intraperitoneally with *B. pseudomallei* K96243 [30]. For the most part, close genetic and antigenic homology means that material from any of the three species can be used to vaccinate against glanders or melioidosis but does not discount the possibility that species-specific antigens might be absent [31,32,33]. 

An effective vaccine would induce high levels of protection and be safe and easy to produce. Live-attenuated vaccines for glanders have been produced and can protect mice from acute glanders [14,34]. The major drawback for live vaccines is that safety and efficacy testing in humans is prohibitive. The numerous antigens present in whole cells are an advantage that subunit vaccines lack. Heat-killed vaccines also contain numerous antigens but are considered too risky for human vaccination. Effective glycoconjugate and nanoparticle-based vaccines have also been produced but may be limited by the minimal amount of antigens that are present in the formulations [35,36,37,38]. An alternative is the production of outer membrane vesicles. Outer membrane vesicles (OMVs) are produced by Gram-negative bacteria under stress conditions such as antibiotic treatment or nutrient deprivation during stationary growth [39]. The outer membrane blebs off as vesicles that contain many proteins and polysaccharides associated with the outer membrane of the bacterium (and less frequently periplasmic, inner membrane and cytoplasmic in decreasing order) but are non-viable [40,41]. OMV producing bacteria can be engineered to optimize protein cargo present, thus allowing a fine-tuning of the immune responses generated. The EMA and FDA approved the vaccine Bexsero, which includes OMVs as adjuvant, for prevention of meningitis in humans [42]. OMVs have previously been used to protect mice against lethal melioidosis [43,44]. In the previously published works OMVs were isolated from virulent *B. pseudomallei.* The use of an OMV based vaccine has not been evaluated for glanders but the requirement for both significant humoral and cell-mediated immunity in protection from glanders makes OMVs an attractive vaccine candidate. OMVs also fulfill the safety aspects associated with future applications involving humans. To meet and exceed these requirements, OMVs can be isolated from biosafe surrogate species. 

In this work, OMVs were isolated from three biosafe source strains and characterized for presence of polysaccharide antigens. The OMVs were used to vaccinate BALB/c mice and levels of OMV-specific antibodies were investigated. The magnitude and nature of antibody responses to *B. mallei* and *B. pseudomallei* cell lysates and opsonophagocytic ability were measured. BALB/c mice were challenged with virulent type strain *B. mallei* China 7 by the intranasal route and vaccine efficacy evaluated as indicated by prolonged survival. Organ bacterial burden in survivors was also determined. C57BL/6 mice were vaccinated with the best candidate identified in BALB/c mice and protection in combination with sterilizing immunity was assessed. This work represents a step forward in the development of a safe and effective vaccine against acute lethal glanders with the indication of chronic glanders prevention in the form of sterilizing immunity.

## 2. Materials and Methods

### 2.1. Bacterial Strains and Culture Conditions

All Select Agent work was carried out in CDC/USDA Tier 1 select agent approved BSL-3 and ABSL-3 facilities at the University of Florida following Tier 1 regulations. All protocols were approved by the Institutional Biosafety Committee prior to implementation under numbers BA-4200 and RD-4158. *B. mallei* strains (China 7, G-2(3), India 86-567-2, Ivan, SAVP1, and Turkey #1; (obtained from the CDC)), *B. pseudomallei* 1026b (CDC/USDA registered in house bacterial inventory) and *B. thailandensis* strains (E555, obtained from Mahidol University; and TxDOH, obtained from CDC) were grown on LB Lennox broth or agar (5 g/L NaCl) (LB, Fisher BioReagents, Pittsburgh, PA, USA) and grown at 37 °C. LB broth was used for liquid growth of all strains. *B. mallei* strains were grown on or in LB media with the addition of 4% (*v*/*v*) glycerol (LB4G). Select Agent excluded strain Bp82 [45] and was grown on LB or TSA with 0.6 mM adenine (Amresco, Solon, OH, USA). The mouse cell line RAW264.7 (American Type Culture Collection, ATCC) was grown in Dulbecco’s Modified Eagle Medium (DMEM)-high glucose + l-glutamine (HyClone) with 10% FBS (HyClone) in 5% CO_2_ at 37 °C. All plastic ware was Corningware with CellBIND surface. Culturing cells was carried out essentially as described previously [46,47,48,49,50,51].

### 2.2. Stains, Western Blots and ELISA

Whole cell lysates were produced by heating equivalent amounts of live bacteria in 1× PBS at 110 °C for 15 min in a digital heat bath. Select-agent bacteria were prepared in the same manner by a validated and verified protocol to ensure sterility. Sample preparation was carried out as previously described with or without Proteinase K digestion depending on the gel [27]. Samples were mixed with 2× LPS lysis/loading buffer prior to running on a gel. SDS-PAGE was carried out using homemade polyacrylamide gels with a 12% resolving gel and a 4% stacking gel. Coomassie blue stains were carried out using standard procedures. Silver stains of SDS-PAGE gels were done according to the manufacturer’s instructions using the Pierce™ Silver Stain Kit (ThermoFisher, Waltham, MA, USA). Colorimetric Western blots were performed by semi-dry electroblotting of SDS-PAGE run gels onto methanol soaked Immobilon P^SQ^ PVDF membranes from Millipore™ (Burlington, MA, USA) or Odyssey nitrocellulose membranes soaked in buffer from LI-COR™ (Lincoln, NE, USA). Blots were washed with 1× PBS, blocked with 1% skim milk in PBS and detected following manufacturer’s instructions with 1-Step™ Ultra TMB-Blotting Solution (ThermoFisher, Waltham, MA, USA). ELISAs were carried out as previously described [50]. Briefly, Immulon 4HBX flat bottom plates were coated with 100 μL antigen at 100 μg/mL suspended in PBS overnight at room temperature. This concentration of antigen was determined to give the best dynamic range in checkerboard optimization ELISAs. The plates were washed three times with PBS/T buffer, blocked with 5% skim milk in PBS/T for 1 h and washed three times with PBS/T buffer again. Detection was carried out by incubating for 1 h with peroxidase conjugated secondary antibodies (Sigma-Aldrich, St. Louis, MO, USA) diluted 1:1000 in blocking solution. After 1 h, wells were washed 3 times with PBS/T then detected with 1-Step™ Ultra TMB-ELISA solution (ThermoFisher, Waltham, MA, USA). The reaction was stopped by adding 1 M H_3_PO_4_ and the absorbance at 450 nm was measured.

### 2.3. Purification of OMVs

OMVs were purified according to Kulp and Kuehn [52] with some modifications. Starter cultures were grown in 10 mL LB or, if Bp82, LB + adenine overnight and used to inoculate 1 L of the same media as a 1:100 dilution. Cultures were grown at 37 °C in an orbital shaker using 4 L Erlenmeyer flasks to late stationary phase. Upon reaching an optical density at 600 nm (OD_600 nm_) of ~4.0 (at 48–72 h) cells were harvested by centrifugation at 8000× *g* for 20 min at 4 °C. The supernatant was passed consecutively through first a 0.45 μm filter unit and then a 0.22 μm unit (Millipore). The filtered supernatant was centrifuged at 38,000× *g* for 2 h at 4 °C. The pellet was resuspended in 1 mL of PBS and 10% was plated for sterility assurance. Samples were submitted to gradient centrifugation in OptiPrep 45–20% (Sigma) at 280,000× *g* for 3 h at 4 °C. Each layer was analyzed by SDS-PAGE and layers containing OMV material (25% and 30% OptiPrep layers) were combined and the samples were lyophilized and dry weights determined. OMV preparations were suspended to 10 mg/mL in PBS and stored at −80 °C for further analysis. 

### 2.4. Transmission Electron Microscopy of OMVs

OMVs were diluted and allowed to dry on a hydrophobic Formva film—coated copper grid and negatively stained with 2% uranyl acetate. For CPS labeling, the primary antibody was the mouse anti-CPS IgG mAb 4C4 (kindly provided by Dr. David AuCoin at University of Nevada, Reno). The secondary antibody was goat anti-mouse IgG (Electron Microscopy Sciences, Hatfield, PA, USA) conjugated to a 12 nm gold particle and negatively stained with 1% uranyl acetate. Images were captured at 100 kV with Hitachi H-7000 transmission electron microscope (Hitachi, Chiyoda, Tokyo, Japan) at the UF Interdisciplinary Center for Biotechnology Research Electron Microscopy Core. 

### 2.5. MALDI-TOF Scans of Purified Lipid A

LPS was isolated from strains using a modified hot-phenol extraction done essentially as described [50,51,53]. Strains were grown on 8–10 plates of TSA or LB-agar (with adenine in the case of Bp82) for 72 h. Lawns were resuspended in PBS and collected in 2 mL gasketed microcentrifuge tubes and heat-killed at 110 °C for 15 min. Phenol was added to the lysed solution to a final concentration of 50%. Samples were dialyzed until free of phenol using tubing with 12–14 kDa molecular weight cutoff against distilled water for 3–5 days. The samples were treated with DNase I for 2 h, RNase h for 2 h, and Proteinase K overnight, then further purified as previously described [53]. Samples were weighed and resuspended in 2 mL of endotoxin free water. For isolation of lipid A, 2 mg of each LPS samples were treated with 1 M acetic acid for 1.5 h on a 100 °C heat-block essentially as previously described [54]. Samples were lyophilized and resuspended in 100 μL of water using a sonicating water bath for 10 min. Lipid As were purified by precipitation with 500 μL of acidified ethanol (1% HCl in 100% ethanol), centrifuged at 12,000× *g* and 4 °C for 10 min, twice washed with non-acidified 100% ethanol, and then dried under vacuum. 

For MALDI-TOF/TOF analysis, dried samples were prepared as previously described [51] in a matrix of 500 mM 2,5-dihydribenzoic acid [54,55]. Mass spectra were acquired with a 4700 MALDI-TOF/TOF analyzer (ABSciex, Framingham, MA, USA) and a Nd:YAG (neodymium-doped yttrium aluminum garnet) laser with a 200-Hz sampling rate. The instrument was operated in the negative ion reflector mode, using a fixed laser intensity of 2500 to accumulate 1000 shots/spectrum across the mass range of 1200–1900 Da. Acquired spectra T2D files were converted to mzXML files and further analyzed on the open source mMass software. Three independently isolated lipid A samples had their mass scans normalized and averaged for the spectrums presented.

### 2.6. Animal Experiments and Ethical Statement

Select Agent animal work was performed in a CDC/USDA Tier 1 approved facility at the University of Florida following Tier 1 regulations. Animal protocols were approved by the Institutional Animal Care and Use Committee at the University of Florida before work began. Female BALB/c mice and C57BL/6 mice between 4 and 6 weeks of age were purchased from Charles River Laboratories (Wilmington, MA, USA). OMV material was diluted to 25 μg/mL in PBS. The prime vaccine doses of 100 μL containing 2.5 μg OMV were inoculated subcutaneously by syringe. Two weeks later the animals were boosted with the same dose and the second boost was given two weeks after that. Two weeks after the last boost mice were tail vein bled for serum and four weeks after the last boost, mice were moved into micro-isolator cages under pathogen-free conditions for challenge at BSL3. The *B. mallei* China 7 inoculate was grown overnight in LB4G media and frozen in 20% glycerol aliquots at −80 °C. Forty-eight hours later, an aliquot was thawed, diluted, and plated for CFU enumeration on LB4G agar medium. Dilution values were determined for the target inoculation of 1.5 × 10^4^ CFU (22 × LD_50_ for BALB/c mice [33] and 17 × LD_50_ for C57BL/6 mice [56]) in 20 μL. Animals were anesthetized with 87.5 mg/kg of ketamine (Patterson Veterinary) of body weight plus 12.5 mg/kg xylazine. Once fully anesthetized, groups of 5 mice (n = 5) were challenged with the 20 μL inoculum by pipetting into the nares of the mouse alternating nostrils until fully inhaled. Mice were observed twice daily for the first 4 days then once daily until the end of the studies. Mice were euthanized when moribund or at the end of the study. 

Organs were processed as follows. The lungs, livers, and spleens from survivors were isolated and organs were homogenized in 5 mL of PBS using a stomacher (Seward, AK, USA). Undiluted and diluted aliquots were plated on LB4G for CFU determination after 48 h growth at 37 °C. Colonies were positively identified as *B. mallei* by testing with the latex agglutination test as previously described [57,58].

### 2.7. Characterization of Antibody Responses

ELISAs for detection of anti-OMV antibody induction in OMV-vaccinated and vehicle only mice were carried out by coating each well overnight with 100 μg/mL of OMV in PBS. This amount of OMV was determined to give us the best dynamic range from optimization ELISAs so that even low level antibody responses could be detected. Serum samples were serially diluted starting at 1:20 for OMV ELISAs in blocking solution and incubated in the plates for 1 h following the protocol above. For ELISAs measuring relative OMV induced anti-*B. mallei* or *B. pseudomallei* antibody responses, 50 ng equivalents (optimally determined by checkerboard assay) of heat-killed *B. mallei* China 7 or *B. pseudomallei* 1026b of known CFU were used to coat plates assuming a single bacterium has a mass of 10^−12^ g. Total bound mouse serum at 1:500 dilution (optimally determined by checkerboard assay) was detected with goat anti-Mouse IgG/IgA/IgM (H + L)-HRP (Thermo Fisher) and developed as described above. Numbers presented are in Absorbance units to allow direct correlation of total antibody production by combined measure of all isotypes.

Characterization of *B. mallei* IgG responses induced by OMV vaccination or vehicle (PBS) only was done using *B. mallei* China 7 lysates as described above but represented as reciprocal endpoint titers because single isotypes were being measured. Serum dilution was started at 1:200 in blocking solution and serially diluted to 1:25,600. Bound IgG1 was detected with goat anti-mouse IgG1-HRP secondary (Thermo Fisher) and bound IgG2a was detected with goat anti-mouse IgG2a-HRP (Thermo Fisher); both at 1:1000 dilution in blocking solution. The endpoint titer was determined as the dilution above the cutoff where the cutoff is three standard deviations above the mean of all PBS serum measurements. If there was no observable reaction at 1:200, a dilution factor of 1:100 was arbitrarily assigned as the cutoff. Results are expressed as the individual reciprocal endpoint titers or as the ratio of the geometric mean reciprocal endpoint IgG1 and IgG2a titers.

### 2.8. Opsonophagocytosis Assay

The opsonophagocytosis assay was performed by diluting *B. mallei* China 7 strain grown in LB4G medium overnight at 37 °C in PBS to an OD_600 nm_ of 1, empirically determined to be ~5 × 10^8^ *B. mallei* CFU. The cultures were then diluted down to 5 × 10^6^ CFU/mL in DMEM or DMEM + 0.2% heat-inactivated (56 °C for 30 min) pooled mouse serum in Hyclone™ Dulbecco’s Modified Eagle Medium (DMEM) (GE Healthcare Life Sciences, Chicago, IL, USA). The dilutions were used to infect RAW264.7 macrophages in 24-well CellBIND plates (Corning, Corning, NY, USA) at an MOI of 10:1. After 1 h, the bacteria-containing medium was removed and the monolayers were washed three times with pre-warmed PBS and fresh DMEM containing 250 μg/mL of kanamycin was added to suppress extracellular bacterial growth. At 2 h post infection the media was removed, monolayers were washed 2 times with PBS and lysed by adding 1 mL of 0.2% Triton-X100 in PBS for 15 min. The lysate was diluted, plated onto LB4G plates and incubated at 37 °C for 48 h to enumerate colonies. Intracellular CFU were calculated and invasion efficiency was determined by dividing the intracellular CFU by the initial number of infecting bacteria. The experiment was carried out in quadruplicate and the numbers represent the average of all four replicates with the error bars representing the SEM. Data shown is representative of two independent experiments.

### 2.9. Statistical Analysis

Significant differences in antibody responses to whole cell lysate and intracellular CFU in the opsonophagocytosis assay between all groups was determined by ordinary one-way ANOVA using Tukey’s multiple comparisons test where ns *p* > 0.05, * *p* ≤ 0.05, ** *p* ≤ 0.01, *** *p* ≤ 0.001, **** *p* ≤ 0.0001. Statistically significant increases in survival were determined by Log-rank (Mantel-Cox) test where * *p* ≤ 0.05, ** *p* ≤ 0.01, *** *p* ≤ 0.001, **** *p* ≤ 0.0001.

## 3. Results

### 3.1. Purification and Characterization of OMVs

OMVs were purified from two *B. thailandensis* strains, E555 and TxDOH. Both strains are known to produce a similar *B. pseudomallei* or *B. mallei* capsular polysaccharide. Strain E555 produces the same O-antigen structures as those found in *B. pseudomallei*. TxDOH harbors a point mutation in *oacA* resulting in a truncated OacA protein. Because OacA is required for 4-*O* acetylation its LPS O-antigen most likely lacks the 4-*O* acetylation of the talose residue similar to the *B. mallei* LPS O-antigen [27]. A biosafe *B. pseudomallei* strain Bp82, an attenuated select agent excluded derivative of *B. pseudomallei* 1026b [45], was also used to isolate OMVs. The previously published methods used to isolate OMVs from bacteria in exponential phase for melioidosis vaccination [43,44] were not ideal in our hands but served as an excellent starting point. In this work a less delicate method involving growth of bacteria to stationary phase, pelleting of the bacteria, two-step filtration, and density gradient centrifugation produced consistent results.

**Figure 1:**
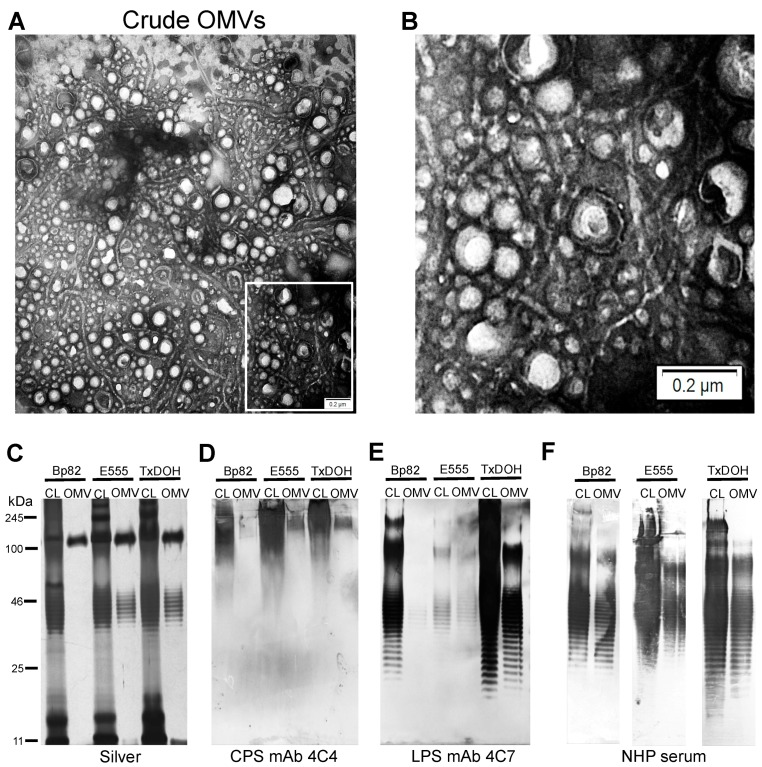
Analysis of outer membrane vesicles (OMVs) and immunogenic polysaccharides. (**A**) Crude OMV negative stained TEM image showing numerous vesicles and flagellar material. (**B**) Zoom image of crude OMV from the area indicated by the white box in (**A**). (**C**) Silver stain of polysaccharide preparations from cell lysates (CL) and OMVs. (**D**) Western blot of the major *B. mallei* capsular polysaccharide (CPS) reveals the high molecular weight polysaccharide is present in all three biosafe strains and their OMVs. (**E**) Western blot of the LPS O-antigen from all three biosafe strains and their OMVs using *B. mallei* O-antigen specific mAb 4C7. (**F**) Reactivity of non-human primate (NHP) serum with cell lysate and OMVs from all three biosafe strains.

**Figure A1:**
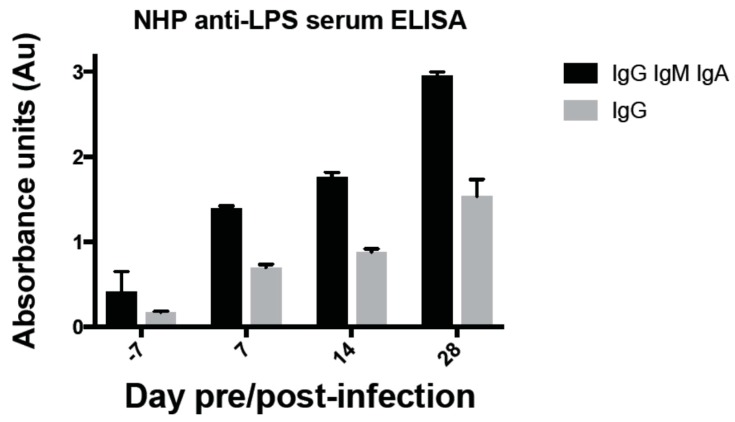
Non-human primate pre/post challenge anti-LPS responses.

**Figure A2:**
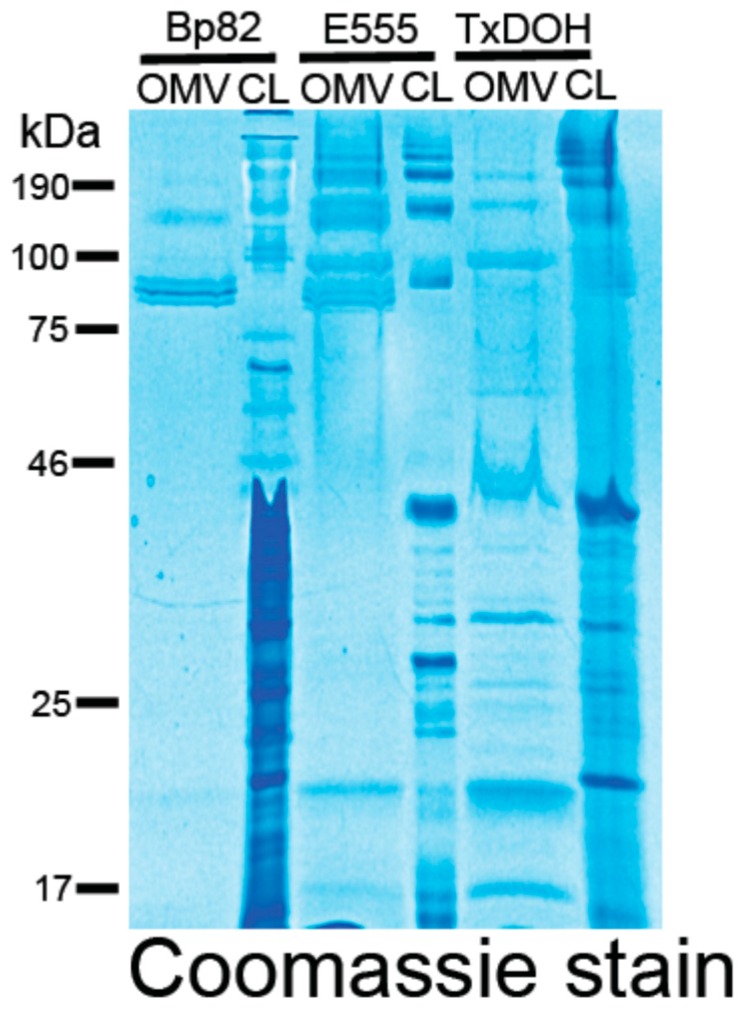
Coomassie stains of OMV preparations compared to cell lysates.

Transmission electron microscopy (TEM) images of the crude OMV preparations show numerous OMVs and flagellar fragments present (Figure 1A,B). Figure 1C–E shows that polysaccharides present in the cell lysates are also present in the OMV preparations. Silver stains, LPS, and CPS Western blots show that the biosafe strain-derived OMV preparations contain the major capsular polysaccharide, CPS, and LPS. Of particular note is the strong signal from the TxDOH O-antigen in Figure 1E, lanes TxDOH, CL and OMV. The 4C7 mAb has a high affinity to the talose residue of the O-antigen without the acetylation [59]. 4 *O*-acetylation of this site, as in Bp82 and E555 LPS, results in reduced binding affinity by mAb 4C7. Serum from a rhesus macaque 28 days post-challenge with aerosolized *B. pseudomallei* strain 1026b shows intense reactivity to mainly the LPS O-antigen in all cell lysates from the OMV parent strains and OMV preparations (Figure 1F). This particular macaque demonstrated very low background levels of anti-LPS specific antibodies prior to aerosol challenge that increased post-exposure, indicative of a highly specific Western blot (Appendix A Figure A1). Coomassie blue staining of the equivalent amounts of OMVs and cell lysates indicate higher molecular weight proteins predominate the Bp82 and E555 OMV preparation while a wider range of proteins are present in the TxDOH OMV preparations (Appendix A Figure A2). 

**Figure 2:**
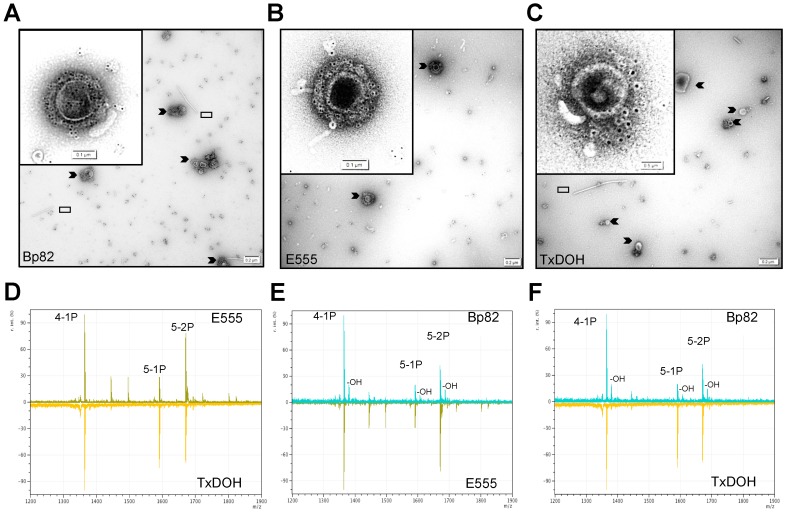
Transmission electron microscopy of capsular polysaccharides and lipid A moieties present in OMV production strains. (**A**–**C**) TEM images of Bp82, E555, and TxDOH-derived OMVs (chevrons), insets are zoomed images of CPS (mAb 4C4 specific immunogold labeling; black dots) in close association with the OMVs. (**D**–**F**), MALDI-TOF/TOF mass scans of lipid A purified from the indicated strains. Flagellar fragments are indicated by caret symbols. (**D**) E555 (top, olive) compared to TxDOH (bottom, gold) lipid A. (**E**) Bp82 (top, cyan) compared to E555 (bottom, olive) lipid A. (**F**) Bp82 (top, cyan) compared to TxDOH (bottom, gold) lipid A. 4-1P, mass peak of tetra-acylated singly phosphorylated lipid A; 5-1P, mass peak of penta-acylated singly phosphorylated lipid A; 5-2P, mass peak of penta-acylated doubly phosphorylated lipid A; -OH, indicates additional hydroxyl groups not present in *B. thailandensis* lipid A moieties.

Further refinement of the association between CPS and the *B. thailandensis* OMV membranes was accomplished by TEM immunogold labeling of CPS using the CPS-specific mAb 4C4 as primary antibody [60]. The black dots surrounding the OMVs indicate large amounts of CPS associated with OMVs from Bp82 and the *B. thailandensis* OMVs (Figure 2A–C insets and chevrons). Visible in these TEM images of the crude preparations are flagellar fragments and OMVs of different diameters. It has been shown that most *B. pseudomallei* LPS are weaker activators of innate immunity [51,61,62] compared to those from *B. thailandensis*. LPS was isolated from the three biosafe strains used in the study and the molecular masses of the hydrolyzed lipid A fragments were measured by MALDI-TOF and compared (Figure 2D–F). The lipid A moieties from E555 and TxDOH have more penta-acylated lipid A than the Bp82 lipid A and do not contain the additional hydroxyl groups that are found in *B. pseudomallei* lipid A, including Bp82. Both characteristics are associated with increased ability to induce innate immunity as compared to less penta-acyl lipid A and presence of hydroxyl groups, as in the Bp82 lipid A. Following density gradient purification TEM analysis of vaccine preparations indicated highly pure OMV free of flagellar contamination. Contribution of flagellar contamination to protective responses was deemed negligible as *B. mallei* is non-motile and previously published data show that including the flagellar component FliC in a glanders nanoparticle vaccine did not enhance post-challenge survival or antibody responses compared to a non-specific TetHc protein component [35]. 

### 3.2. Strong Antibody Responses Are Generated by OMV Vaccination

**Figure 3:**
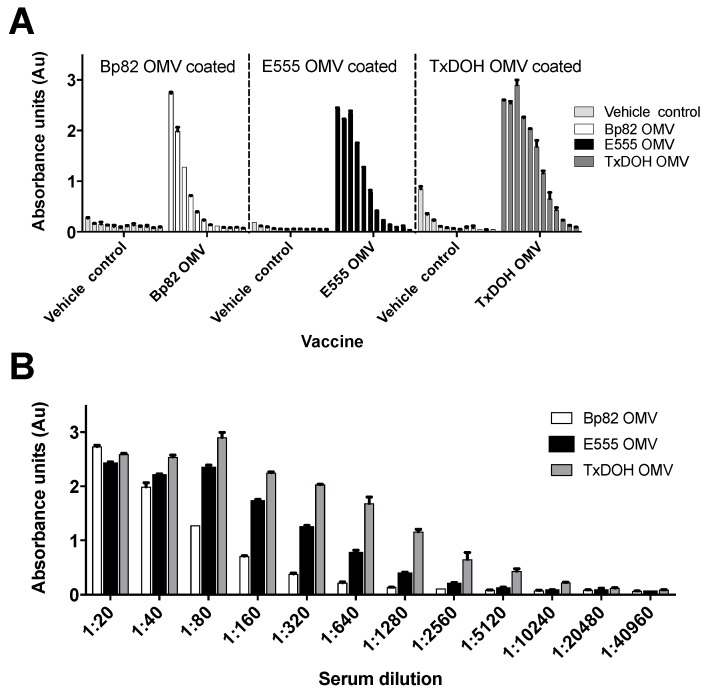
Stronger OMV specific antibodies are produced after vaccination with *B. thailandensis*-derived OMVs. (**A**) Average reactivity of serially diluted serum from OMV-vaccinated mice to the immunogenic material is specific compared to serum from vehicle control-vaccinated mice. (**B**) Endpoint serum dilution compared between OMV vaccinated groups measured by ELISA and represented as absorbance units at 450 nm.

Following the prime-boost-boost vaccination regimen with PBS and the different OMVs, mouse serum was collected and ELISAs were run to compare relative antibody responses to the vaccination material (Figure 3A,B). PBS control serum had very low reactivity to each material while vaccinated mice had high levels of serum antibody to each respective OMV preparation. The reciprocal endpoint titers that were three standard deviations above the PBS vaccinated mean for Bp82, E555, and TxDOH vaccinated mouse serum were 320, 2560, and 1280, respectively. This indicated an eight-fold higher antibody response generated by E555 OMVs and four-fold higher response by TxDOH OMVs compared to Bp82 OMVs. 

### 3.3. Characterization of B. mallei Specific Antibody Responses to OMVs

**Figure 4:**
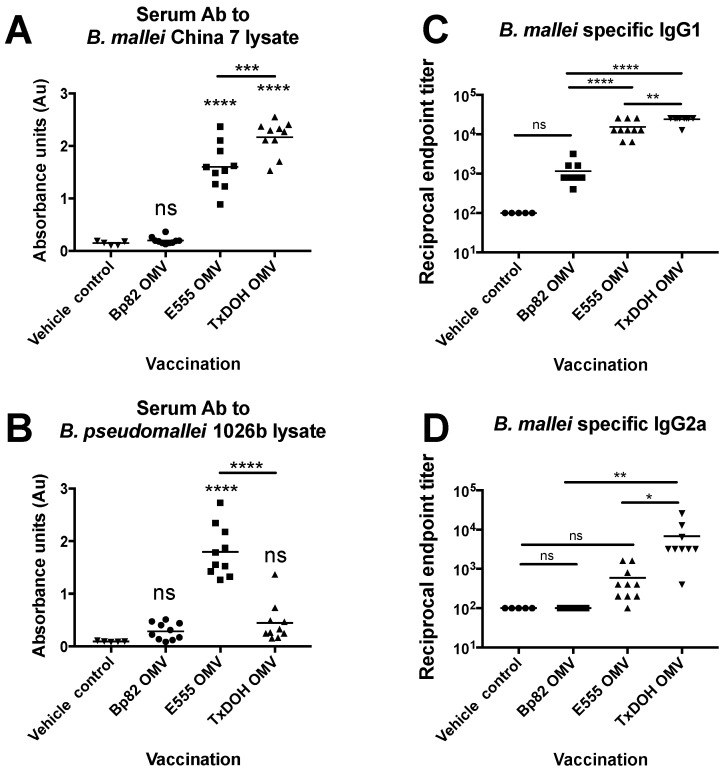
Biosafe strain-derived OMVs produce *B. mallei* and *B. pseudomallei* specific serum antibody responses. (**A**) Serum antibody responses in vehicle only or OMV-vaccinated mice (PBS n = 5, all others n = 10) to *B. mallei* China 7 lysate. (**B**) Serum antibody responses in vehicle only or OMV-vaccinated mice to *B. pseudomallei* 1026b lysate (PBS n = 5, all others n = 10). Measured by ELISA and represented as absorbance units at 450 nm. (**C**,**D**) Serum IgG1 and IgG2a responses in vehicle only or OMV-vaccinated mice (PBS n = 5, all others n = 10) to *B. mallei* China 7 lysate expressed at reciprocal endpoint titer. One-way ANOVA was used to determine statistical significance, ns *p* > 0.05, * *p* ≤ 0.05, ** *p* ≤ 0.01, *** *p* ≤ 0.001, **** *p* ≤ 0.0001.

To compare the magnitude of *B. mallei* cross-reactive antibody production, serum samples were run against heat-killed lysate from *B. mallei* China 7 and bound mouse immunoglobulin was measured. It was found that subcutaneous vaccination using TxDOH OMVs produced the strongest anti-*B. mallei* antibody response, significantly more than E555 OMVs (Figure 4A). Both *B. thailandensis*-derived OMVs generated significantly higher anti-*B. mallei* antibody responses than Bp82 OMVs, which were not significantly different than PBS vaccinated mouse sera. Specificity of the antibody response to *B. mallei* was measured by running the same samples against *B. pseudomallei* 1026b heat-killed lysate (Figure 4B). In contrast to the observation in Figure 4A, anti-*B. pseudomallei* antibody responses generated by TxDOH OMVs were not significantly different than those in PBS vaccinated mice. E555 OMVs generated high levels of anti-*B. pseudomallei* antibody responses in addition to anti-*B. mallei* antibody responses. 

Anti-*B. mallei* serum IgG1 and IgG2a levels were measured by determining the reciprocal end-point titer of reactivity to heat-killed *B. mallei* China 7 lysate (Figure 4C,D). The IgG results followed a similar trend as with the poly-Ig. TxDOH vaccinated mice had significantly higher amounts of IgG1 and IgG2a in serum than all other vaccinated mice. The mean reciprocal IgG1 titer of E555 OMV vaccinated mice following the subcutaneous vaccination regimen was significantly higher than Bp82 OMV or PBS vaccination. These values were used to calculate the IgG1/IgG2a ratio (Table 1). If the ratio is below or close to one the antibody response can be more indicative of a cell mediated antibody response where IFN-γ suppresses IgG1 production and increases IgG2a production, while if much higher than 1, a type 2 antibody response predominates [63]. Vehicle only vaccinated mice have no detectable IgG1 or IgG2. The order of ratio for OMV vaccinated mice is as follows TxDOH < Bp82 << E555, (4.09, 9.84, and 34.30, respectively). All ratios were above one, indicating a type 2 antibody response is produced in mice following vaccination with all OMV preparations. 

**Figure 5:**
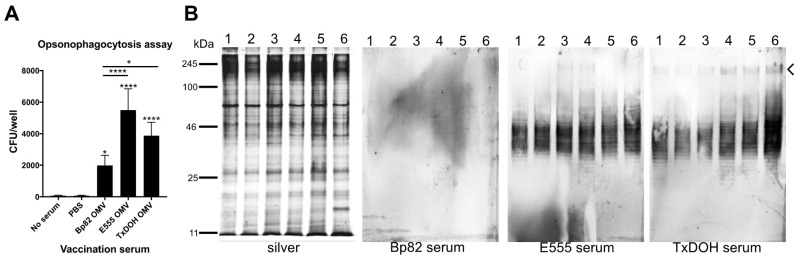
Opsonophagocytosis of *B. mallei* by RAW264.7 murine macrophages and serum reactivity to other *B. mallei* strains. (**A**) Number of intracellular *B. mallei* following opsonization with OMV-vaccinated mouse serum by RAW264.7 murine macrophages. Significance determined by one-way ANOVA, * *p* ≤ 0.05, **** *p* ≤ 0.0001. (**B**) Silver stain and Western blots of reactivity between six different *B. mallei* strain lysates and pooled mouse serum from the indicated OMV-vaccinated mouse (n = 10) groups. Lanes 1–6, *B. mallei* strains China 7, G-2(3), India 86-567-2, Ivan, SAVP1, and Turkey #1, respectively. Caret indicates high molecular weight CPS reactivity.

The ability of the various serum samples to promote opsonophagocytic uptake of *B. mallei* China 7 by murine macrophage cell line RAW264.7 was assessed (Figure 5A). Intracellular bacteria numbers were enumerated and showed that pooled and heat-inactivated mouse serum from OMV vaccinated mice significantly increased opsonophagocytosis several magnitudes higher than pooled PBS vaccinated mouse serum or no serum controls as evidenced by the increase in intracellular CFU from ~100 to several thousand CFUs. Calculated invasion efficiencies (intracellular CFU/total inoculated CFU) were as follows; No serum and PBS: 3.1 × 10^−5^, Bp82: 8.2 × 10^−4^, E555: 2.28 × 10^−3^, and TxDOH: 1.60 × 10^−3^. Mouse serum from each of the three vaccinated mouse groups was also used to probe SDS-PAGE run cell lysates from five *B. mallei* isolates in addition to *B. mallei* China 7 (Figure 5B). Lanes 1–6 coincide with *B. mallei* strains China 7, G-2(3), India 86-567-2, Ivan, SAVP1, and Turkey #1; (obtained from the CDC). Cell lysates were run on SDS-PAGE followed by silver staining or Western blotting. Blotting the lysates with Bp82 OMV vaccinated mouse serum shows the weak interaction with the *B. mallei* lysates. The E555 and TxDOH OMV vaccinated mouse sera show strong reactivity to the LPS O-antigens of all isolates and even to the high molecular weight CPS (indicated by chevron). The TxDOH OMV vaccine even appears to induce more CPS reactive antibodies than the E555 OMV vaccine when comparing the Western blots between the two. 

### 3.4. Ability of OMV Vaccines to Protect from Acute and Chronic Inhalational Glanders in BALB/c Mice

**Figure 6:**
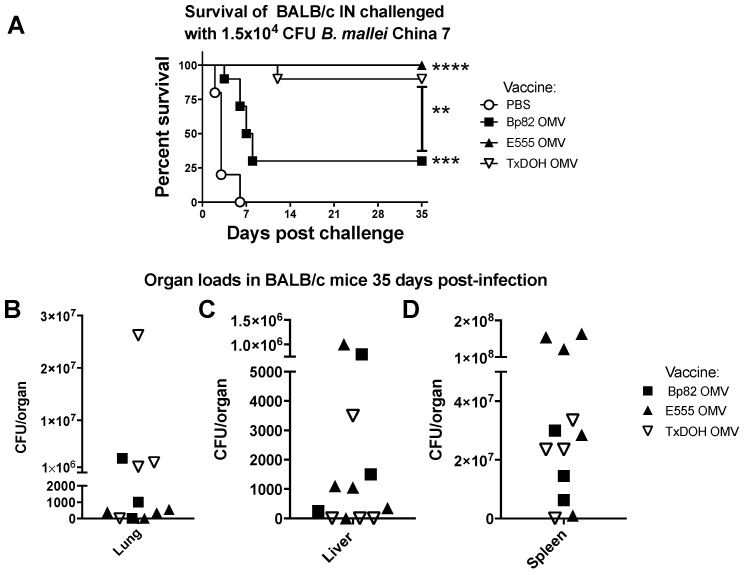
OMV vaccination protects BALB/c mice from acute lethal glanders following intranasal inoculation. (**A**) Survival to 35 days of 4–6 weeks old BALB/c mice intranasally challenged with 1.5 × 10^4^ CFU *B. mallei* China 7 after prime-boost-boost vaccination with the Bp82-, E555-, TxDOH-derived OMV (each group n = 10) or PBS (n = 5). Statistically significant increases in survival compared to PBS or each other as indicated were determined by the Log-rank (Mantel-Cox) test where ** *p* ≤ 0.01, *** *p* ≤ 0.001, **** *p* ≤ 0.0001. Bacterial loads in the lungs (**B**), livers (**C**), and spleens (**D**) of 35-day survivors. In panels (**B**–**D**) each symbol equals one mouse.

The ability of OMVs to vaccinate and protect mice from lethal glanders up to the critical 30-day mark was assessed next (Figure 6). Groups of 10 vehicle only or OMV-vaccinated BALB/c mice (prime and boosted twice at two-week intervals) were challenged 4 weeks after the final boost with an acutely lethal intranasal dose of 1.5 × 10^4^ CFU *B. mallei* China 7 and survival proportions were recorded (Figure 6A). Mice receiving a mock vaccine of PBS succumbed within six days to acute infection. Two-thirds of Bp82 OMV vaccinated mice succumbed during the same time period with the remaining mice surviving out to the 35-day study, endpoint resulting in a significant increase in survival compared to PBS vaccinated mice. Mice receiving either the E555 or TxDOH OMV vaccines were protected from acute lethal glanders with only one TxDOH OMV-vaccinated mouse succumbing at 12 days post-infection (dpi) and 100% of E555 OMV-vaccinated mice surviving to the 35-day study endpoint. Statistical significance of the E555 and TxDOH OMV vaccinated groups compared to the vehicle only group with the number of animals used has greater than 95% power but decreases to 50% power when comparing the vehicle only and the Bp82 OMV vaccinated groups.

However, mice appeared slightly emaciated so three Bp82 OMV, five E555 and four TxDOH mice were humanely sacrificed to evaluate bioburden in the lungs, livers, and spleens (Figure 6B–D). Organ bacterial burdens were determined and high CFU numbers were found in the spleens of all survivors tested indicating these vaccines did not achieve effective immunity in BALB/c mice. Gross pathology of spleens revealed numerous abscesses and splenomegaly. Each group had at least one mouse that had no detectable bacteria in the lungs. Three TxDOH-vaccinated mice and one E555-vaccinated mouse had no detectable bacteria in their livers and one mouse from each had cleared the bacteria in the lungs and livers. Perhaps this is due to high levels of neutralizing antibodies produced in the Th2 dominating immune response we measured in the BALB/c mouse background that then drives intracellular infections that occur in the spleen. 

**Figure 7:**
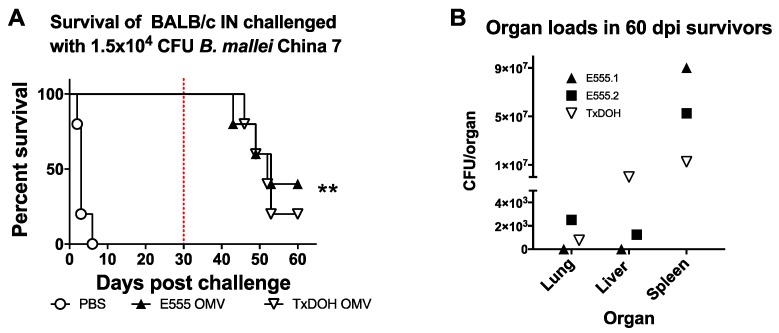
OMV vaccination protects BALB/c mice from chronic glanders following intranasal inoculation. (**A**) 4–6 weeks old BALB/c mice intranasally challenged with 1.5 × 10^4^ CFU *B. mallei* China 7 after prime-boost-boost vaccination with E555-, TxDOH-derived OMV or PBS (each group n = 5) were observed for survival out to 60 days. Statistically significant increases in survival compared to PBS vaccinated mice were determined by the Log-rank (Mantel-Cox) test where ** *p* ≤ 0.01. The red-dashed line indicates an important milestone of 30 days post-infection. (**B**) Bacterial loads in the lungs, livers, and spleens of 60-day survivors.

Protection beyond 35 days is also desirable because chronic infections can lead to death well beyond 35 days. Groups of 5 additional BALB/c mice receiving PBS, E555 OMV or TxDOH OMV vaccines were observed for survival following intranasal challenge with 1.5 × 10^4^ CFU of *B. mallei* China 7 out to 60 dpi. Both vaccines afforded significant protection from acute lethal glanders to all mice and all survived out past 30 dpi (Figure 7A), reiterating the findings in Figure 6A. Starting at 43 dpi, vaccinated mice began to succumb to chronic glanders infection. By the 60-day study endpoint, 2/5 E555 OMV vaccinated mice survived and only 1/5 from the TxDOH vaccinated group. The overall increase in survival in vaccinated groups was highly significant compared to the vehicle only vaccinated controls. Groups of five mice gave 80% power to our statistical significance at day 50 post-infection. Survivors were humanely sacrificed and bacterial organ loads were determined (Figure 7B). Splenic loads were very high in the three survivors with greater than 1 × 10^7^ CFU present. One E555 OMV vaccinated mouse had no detectable CFU in the lung or liver and the other also had relatively low levels. Gross pathology of the organs showed chronic splenic involvement with advanced abscess formation, substantial splenomegaly, and tight splenic adherence to the intra-abdominal wall, requiring extensive surgical removal. 

### 3.5. Ability of B. thailandensis E555 OMV Vaccine to Protect from Acute Lethal Glanders in C57BL/6 Mice

**Figure 8:**
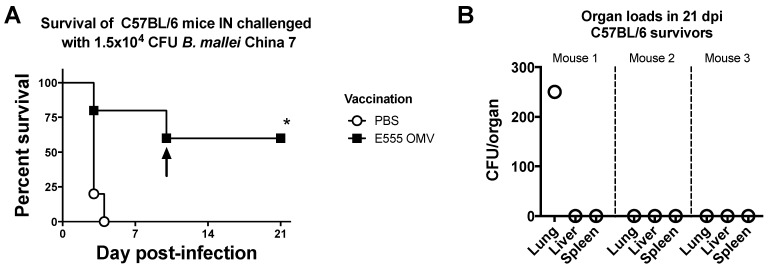
OMVs derived from *B. thailandensis* E555 protect C57BL/6 mice from acute lethal glanders and can provide sterilizing immunity following intranasal inoculation. (**A**) 4–6 weeks old C57BL/6 mice intranasally challenged with 1.5 × 10^4^ CFU *B. mallei* China 7 after prime-boost-boost vaccination with E555-derived OMV or PBS (each group n = 5) were observed for survival out to 21 days. Statistically significant increases in survival compared to PBS vaccinated mice were determined by the Log-rank (Mantel-Cox) test where * *p* ≤ 0.05. Arrow indicates mouse was euthanized for neurological symptoms. (**B**) Organ loads of lungs, livers, and spleens of 21-day survivors showing sterilizing immunity in two mice and close to sterilizing immunity in the third.

The OMV vaccine produced from *B. thailandensis* E555 was chosen as the best candidate to assess in the well-characterized C57BL/6 mouse because of the high levels of antibodies specific to both *B. mallei* China 7 and *B. pseudomallei* 1026b lysates (Figure 4). Five C57BL/6 mice were vaccinated with E555 OMVs using the same regimen as in the studies with BALB/c mice, then challenged with the same acutely lethal dose of *B. mallei* China 7 (17 × LD_50_ in C57BL/6 mice, and observed for survival (Figure 8A). All PBS vaccinated control mice died by 4 dpi. E555 OMV vaccination provided significant protection from acute lethal glanders. One mouse succumbed to acute glanders on day 3. A second mouse with neurological symptoms that otherwise appeared healthy was humanely euthanized at 10 dpi. The other three mice survived to day 21, the study endpoint. The three survivors were sacrificed and bacterial burden was assessed by organ load determination. Figure 8B shows that mouse 1 had ~250 CFU of *B. mallei* in the lung and had no measurable bacterial CFU in the livers or spleen. The other two mice had no measurable *B. mallei* CFU in lungs, livers, or spleens, thus indicating efficient bacterial clearance was achieved in 40% of mice (2/5), and nearly achieved in the third mouse that had bacteria detected in the lungs but not the liver or spleen. Establishment of a stronger Th1 response in the C57BL/6 background could explain why the surviving mice had cleared most of the bacteria. C57BL/6 mice are more resistant to infection resulting in a lower LD_50_ equivalency challenge dose compared to the BALB/c mice but was still well within a high-dose LD_100_ range. Future studies will look at the T-cell responses to OMV vaccination in the C57BL/6 mice.

## 4. Discussion

*B. mallei* is a lesser known but no less notorious biological warfare agent. The disease it causes is difficult to prevent due to high pathogenicity and the intracellular nature of the organism. Development of a vaccine for glanders has been focused mainly on subunit vaccines that have had varying degrees of effectiveness [35,36,64] and live-attenuated vaccines [14,33,34] that face many hurdles to human use. Through our use of biosafe strain-derived OMVs to generate protective immune responses we have circumvented both issues. We showed that the OMVs from all strains have CPS associated with them. Bp82 OMVs generated very poor anti-*B. mallei* antibody levels compared to either *B. thailandensis*-derived vaccine and could explain why the Bp82 OMVs provided significantly less protection. The success of other OMV vaccines, including the melioidosis OMV vaccine, has been attributed to strong type 2 antibody responses [44,65,66]. In this study, strong antibody responses were observed following vaccination with OMVs particularly towards the LPS O-antigen and CPS. Other works have shown high IgG and IgM responses are generated by mice vaccinated with the CPS from *B. thailandensis* E555 [30]. The higher immunogenicity of lipid A in *B. thailandensis* LPS may contribute to better memory responses by inducing stronger local inflammation that attracts more antigen presenting cells. Lipid A and liposomes containing immunogenic lipid A are known to be potent adjuvants for vaccines and help induce Th1 responses [67,68]. Figure A2 also demonstrated that the protein content of the OMVs vary substantially. It appears that the OMVs from *B. thailandensis* contain higher quantities of common proteins and several proteins that are not found in *B. pseudomallei*-derived OMVs. These may have been protective antigens that generated a cell-mediated response leading to protection in the vaccination model utilized. The identities of these proteins will be determined in future analysis. 

The BALB/c mouse model is generally accepted to be an appropriate and moderately sensitive infectious disease model for melioidosis and glanders [69,70]. Despite this we were able to elicit significant protection to acute lethal challenge of 22 × LD_50_ and even to chronic lethal glanders. However, immunity was not sterilizing and protection from chronic glanders was incomplete; potentially due to the enhanced type 2 antibody responses that dominate the BALB/c mouse immune response [71,72,73,74]. Chronically infected BALB/c survivors had nearly cleared bacteria from the lungs but the high burden in the spleen indicated a failure of proper cell-mediated immune responses in this model. Since C57BL/6 mice are generally more resistant to *B. mallei* infection and generate a more Th1-skewed response we wanted to test our best vaccine candidate in this inbred mouse line. The decision to move forward with E555 OMVs was two-fold: 1. It was able to generate high amounts of antibodies to both *B. mallei* and *B. pseudomallei* lysate. 2. It provided 100% protection from acute lethal glanders in BALB/c mice. Testing in C57BL/6 mice showed significant protection from acute lethal glanders and 2/5 mice had no measurable bacteria in the target organs. The livers and spleens of 3/5 mice were free of detectable bacteria.

The OMV vaccines generated in this work are excellent candidates for a human vaccine. They are safe, easy, and cheap to produce. These strains are not considered human pathogens so infectivity is not a worry and biosafety concerns associated with growth volume are considerably reduced. The one-liter growth preparation used in this study typically results in 100–200 mg of lyophilized OMV material. For perspectives sake, that is enough material to vaccinate ~13,000–26,000 mice with the full three-dose vaccine regimen. Human doses are surely higher but even then, tangential flow filtration can be added to the workflow for high volume scale-up if desired. Another bonus is stability. The OMVs can be lyophilized, frozen, thawed, and rehydrated while retaining their immunogenic properties (this work) and can also provide a protective environment for active enzymes [75]. 

Future directions include increasing the dose of OMV vaccine given. Our dose of 2.5 μg is a modest dose and could be increased to see if protection from a higher dose challenge can be achieved. We also found a sizeable portion of the antibody response was to the LPS O-antigen. It is possible that the numerous repetitive T-cell independent antigens present in LPS are masking protein antigens important for generating cell-mediated immune responses. Eliminating these components and observing the effect on antibody production or survival in mice is another way we can enhance effective T-cell memory responses. Future work in this direction would lead to greater understanding of protective cell-mediated Th1 responses generated in both BALB/c and C57BL/6 glanders models. We would also like to test its potential as a divalent vaccine by assessing ability to prevent acute lethal melioidosis in addition to glanders. Since high levels of anti-*B. pseudomallei* antibodies were observed, the E555 OMV vaccine could theoretically be used to vaccinate against both glanders and melioidosis. Vaccine formulations in this work did not include any type of licensed approved adjuvant or material beyond what is present in the PBS OMV suspension. Future formulations incorporating vaccine adjuvants such as CpG or poly(I:C) for stimulation of Th1 responses could lead to a higher likelihood of complete immunity. 

## 5. Conclusions 

In conclusion, we believe this is a step forward in vaccine development against glanders and for many of the reasons described above, biosafe strain-derived OMVs represent good candidates for targeted research and development.

## Figures and Tables

**Table 1:** OMV immunizations induce *B. mallei* specific IgG1 and IgG2a responses.

Antigen	Group	Geometric Mean Titer *^1^*		
		IgG1	IgG2a	IgG1/IgG2a Ratio
*B. mallei* China 7 lysate	PBS	100	100	1.00
	Bp82 OMV	984	100	9.84
	E555 OMV	13718	400	34.30
	TxDOH OMV	23702	5796	4.09

*^1^* The geometric mean of the reciprocal endpoint titers with the IgG1/IgG2a ratio of the values indicated for each group.
